# The role of baseline BLyS levels and type 1 interferon-inducible gene signature status in determining belimumab response in systemic lupus erythematosus: a post hoc meta-analysis

**DOI:** 10.1186/s13075-020-02177-0

**Published:** 2020-05-04

**Authors:** Christel Wilkinson, Robert B. Henderson, Angela R. Jones-Leone, Shaun M. Flint, Mark Lennon, Roger A. Levy, Beulah Ji, Damon L. Bass, David Roth

**Affiliations:** 1 GSK, Stevenage, Herts UK; 2 GSK, Upper Providence, PA USA; 3 GSK, Uxbridge, Middlesex UK; 4 GSK, Philadelphia, PA USA

**Keywords:** Systemic lupus erythematosus, B lymphocyte stimulator, B cell activating factor, Belimumab, Interferon, Messenger RNA

## Abstract

**Background:**

Elevated B lymphocyte stimulator (BLyS) levels in patients with systemic lupus erythematosus (SLE) correlate positively with disease activity; BLyS expression is directly linked to interferon (IFN) pathway activation. This post hoc meta-analysis of BLISS-52 and BLISS-76 explored the relationship between baseline BLyS mRNA/protein levels and/or type 1 IFN-inducible gene signature (IFN-1) and responses to the BLyS-targeting monoclonal antibody belimumab in SLE.

**Methods:**

In BLISS-52 and BLISS-76, patients with autoantibody-positive SLE and a SELENA-SLEDAI score ≥ 6 and receiving stable standard SLE therapy were randomised to intravenous belimumab 10 mg/kg or placebo, plus standard of care (SoC), for 52 or 76 weeks. For this post hoc meta-analysis, patients with an appropriate mRNA sample were stratified by BLyS mRNA expression (tertiles: high/medium/low; revised quantiles: high/low), IFN-1 mRNA expression (high/low) and BLyS protein level (high/low). Co-primary endpoints were correlation between baseline BLyS and IFN-1 mRNA levels and SLE Responder Index (SRI)4 response at week 52 within BLyS/IFN-1 subgroups. Secondary endpoints included time to first severe SELENA-SLEDAI Flare Index (SFI) flare.

**Results:**

Of 554 patients included in this analysis, 281 had received belimumab and 273 had received placebo. Baseline BLyS and IFN-1 mRNA levels were highly correlated (Spearman’s rank correlation coefficient 0.7799; 95% confidence interval [CI] 0.7451, 0.8106; *p* < 0.0001). The proportion of SRI4 responders was higher with belimumab versus placebo in all subgroups, but the difference reached statistical significance in the medium BLyS mRNA tertile (odds ratio [OR] 2.17; 95% CI 1.16, 4.04; *p* = 0.0153), high BLyS mRNA quantile (OR 1.58; 95% CI 1.02, 2.44; *p* = 0.0402), high IFN-1 mRNA (OR 1.58; 95% CI: 1.08, 2.31; *p* = 0.0186) and high BLyS protein (OR 3.57; 95% CI 1.63, 7.83; *p* = 0.0015) subgroups only. The risk of severe SFI flare was significantly lower with belimumab than placebo in the high BLyS mRNA quantile (hazard ratio [HR] 0.59; 95% CI 0.36, 0.97; *p* = 0.0371) and high BLyS protein (HR 0.39; 95% CI 0.19, 0.79; *p* = 0.0090) subgroups.

**Conclusions:**

This post hoc meta-analysis demonstrated a tendency towards improved response to add-on intravenous belimumab 10 mg/kg versus SoC alone in patients with high baseline BLyS protein and IFN-1 mRNA levels and medium/high BLyS mRNA levels.

## Background

Systemic lupus erythematosus (SLE) is a chronic autoimmune disorder that affects a variety of organ systems and markedly impairs health-related quality of life [1–3]. The course of SLE is characterised by periods of disease flare and remission, with treatment aiming not only to minimise disease activity, but also to reduce the risk of flares [4]. Patients with autoimmune diseases such as SLE often have increased blood levels of B lymphocyte stimulator (BLyS), also known as B cell activating factor [5–7], a member of the tumour necrosis factor ligand superfamily that promotes the survival of B lymphocytes, inhibits B cell apoptosis and is involved in the differentiation of B cells into immunoglobulin-producing plasma cells [8–11]. Elevated levels of BLyS positively correlate with elevated autoantibody levels, serum immunoglobulin G and disease activity [5–7, 12].

Belimumab is a recombinant, human, immunoglobulin G1 lambda monoclonal antibody that binds soluble BLyS protein and inhibits its biological activity [13, 14]. Belimumab International Systemic Lupus Erythematosus (BLISS)-52 and BLISS-76 were randomised, double-blind, placebo-controlled, multicentre, phase 3 studies of intravenous (IV) belimumab plus standard of care (SoC), with similar study designs, conducted in patients with active SLE in spite of SoC treatment [15, 16]. Results of these studies showed that treatment with belimumab 10 mg/kg IV every 2 weeks for 3 doses and every 4 weeks thereafter was effective in significantly reducing disease activity as measured by the Systemic Lupus Erythematosus Responder Index (SRI) at week 52, the primary efficacy endpoint of both trials.

Data from these trials also suggested that belimumab treatment may be associated with reduced steroid use and reduced risk of severe flare in this population. Interestingly, the difference in the proportion of SRI responders for belimumab versus placebo at week 52 was more evident in patients with a high serum BLyS protein level (≥ 2 ng/ml) at baseline when compared with the total population or those with a BLyS level < 2 ng/ml [17], suggesting that BLyS protein may be a good predictor of response to belimumab. Notably, BLyS messenger ribonucleic acid (mRNA) has shown stronger correlation than BLyS protein to SLE disease activity, suggesting that BLyS mRNA may be a better predictor of response to belimumab than BLyS protein [18, 19]. Furthermore, it has previously been reported that BLyS expression is directly linked to and regulated by interferon (IFN) signalling [20], and improved responses to IFN pathway inhibitors have been seen in patients with SLE and high IFN levels compared with low IFN in early SLE trials [21].

Examining the relationship between the expression of type I IFN-regulated genes (IFN signature) and response to belimumab in the phase 3 trial sample sets could potentially help to clarify the role of the biomarkers in identifying patient subgroups that will respond well to belimumab treatment. As such, a post hoc analysis of data from BLISS-52 and BLISS-76 was conducted in a subset of patients with SLE from whom RNA samples had been collected and stored. The objectives of this study were to evaluate the efficacy of belimumab versus placebo among patients with SLE stratified by whole-blood BLyS mRNA/protein levels and by type 1 IFN-inducible gene signature (IFN-1) status at study baseline.

## Methods

### Post hoc analysis design

This study (GSK study 208651) was a post hoc meta-analysis of the two phase 3 studies of IV belimumab: BLISS-52 (NCT00424476) and BLISS-76 (NCT00410384), in which patients received belimumab 10 mg/kg IV plus SoC or placebo plus SoC for 52 or 76 weeks, respectively. Full details of the original studies have been published elsewhere [15, 16]. This analysis was performed to explore the efficacy of belimumab 10 mg/kg compared with placebo when patients were stratified by whole-blood BLyS mRNA delta cycle threshold (CT; expression) levels (tertiles: high [> 0.650], medium [− 0.061 to 0.650], low [<− 0.061]; binary: high [≥0.0382], low [< 0.0382]) and protein levels (high [≥ 2 ng/mL], low [< 2 ng/mL]) and by IFN-1 mRNA status (high [≥ 1.09], low [< 1.09]) at study baseline.

### Participants

The original study populations included patients with SLE who were autoantibody-positive (antinuclear antibody titre ≥ 1:80 and/or anti-double-stranded deoxyribonucleic acid ≥ 30 IU/mL) with a Safety of Estrogen in Lupus Erythematosus National Assessment-Systemic Lupus Erythematosus Disease Activity Index (SELENA-SLEDAI) score ≥ 6 who had been receiving stable standard SLE therapy for ≥ 30 days prior to enrolment [15, 16]. Patients with severe active lupus nephritis (> 6 g/24 h proteinuria or > 2.5 mg/dL serum creatinine) or severe active central nervous system SLE were excluded. The gene expression population used for this post hoc analysis was defined as all randomised patients who received at least one dose of belimumab 10 mg/kg or placebo during the original studies and who had an mRNA sample that passed quality control. A group of 25 healthy volunteers also provided mRNA samples, which were used to determine a typical distribution of BLyS and IFN-1 mRNA levels.

### Samples

RNA from BLISS-52 and BLISS-76 participants and healthy volunteers was reverse transcribed to c-deoxyribonucleic acid (Applied Biosystems) prior to quantitative polymerase chain reaction measurements in triplicate by Taqman assay (Life technologies) on a Fluidigm BioMark platform (Expression Analysis, Q2 solutions). Outlier replicate CTs were excluded prior to generation of a μCT per gene/sample. Expression was normalised against a panel of housekeeping genes (*ActB*, *GAPDH*, *18S*, *PPIA*, *HPRT1*, *RPLPO* and *B2M*) to generate a ΔμCT for *TNFSF13B* (BLyS) and individual IFN signature genes, *IFI27*, *IFI44*, *IFI44L* and *RSAD2.* The IFN-1 signature ΔΔμCT for each gene and sample was generated by normalisation to the pooled healthy samples; the median of the ΔΔμCT was the IFN-1 signature score for each sample.

BLyS protein data was analysed for the patients from whom BLyS mRNA was assessed.

### Endpoints

There were two co-primary endpoints: the correlation between baseline BLyS mRNA levels and baseline IFN-1 mRNA status in the entire study population, and the SRI4 response at week 52 within BLyS mRNA/protein levels and IFN-1 status subgroups. An SRI4 responder was defined as having a ≥ 4-point reduction from baseline in SELENA-SLEDAI score and no worsening in Physician Global Assessment (PGA) score from baseline (worsening defined as an increase of ≥ 0.3 points) and no new British Isles Lupus Assessment Group of SLE Clinics (BILAG) A organ domain score, or two new BILAG B organ domain scores compared with baseline.

Secondary endpoints for this analysis included SRI4 response over time, time to first severe flare, more extreme SELENA-SLEDAI response (SRI4 responder and [SELENA-SLEDAI total score ≤ 4 or clinical SELENA-SLEDAI total score ≤ 2]) and SRI8 response (modifying the SRI4 response to require an ≥ 8-point reduction from baseline in SELENA-SLEDAI score) in relation to baseline BLyS mRNA and IFN-1 mRNA status subgroups.

Gene expression data were used to investigate the distribution of baseline BLyS mRNA levels, IFN-1 mRNA status and BLyS protein levels. Additionally, the correlation between BLyS protein and mRNA levels at baseline was assessed.

Safety outcomes were not assessed in this post hoc analysis.

### Analyses and statistics

Data are presented for BLyS mRNA tertiles (high, medium, low) and for a revised binary stratification (high, low). The BLyS mRNA tertiles were protocol-defined and created using the 33.33rd and 66.67th percentiles of the quantitative polymerase chain reaction BLyS mRNA delta CT distribution data (Additional file 1: Supplementary Figure S1A), based on a previous publication that demonstrated a prognostic effect of BLyS mRNA levels in patients with SLE [19]. However, as BLyS mRNA tertile stratification findings did not align with previous results, a revised binary stratification of high and low was also implemented based on the distribution of BLyS mRNA delta CT levels for 24 healthy volunteers (Additional file 1: Supplementary Figure S1B). Data from one outlier were excluded as the value for that particular subject did not align with the data from the other 24 volunteers. High and low IFN-1 status subgroups were determined according to IFN-1 mRNA expression (defined as the median mRNA level of *IFI27*, *IFI44*, *IFI44L* and *RSAD2*) in each sample, with a high/low stratification implemented using the trough in the bimodal distribution of IFN-1 as the cut-off (Additional file 1: Supplementary Figure S1C). BLyS protein level subgroups (high, low) (Additional file 1: Supplementary Figure S1D) were defined based on a previous publication that demonstrated a correlation between BLyS protein levels and response to belimumab in patients with SLE [17].

All post hoc analyses were conducted in the gene expression population. A logistic regression model was used to estimate the odds of an SRI4 response for belimumab versus placebo for the BLyS mRNA/protein and IFN-1 mRNA subgroups and overall, with variables of the treatment group, study, baseline SELENA-SLEDAI score (≤ 9 versus ≥ 10), baseline proteinuria levels (< 2 g/24 h versus ≥ 2 g/24 h equivalent) and race (African descent or Native American descent versus other), and the BLyS subgroup or IFN-1 mRNA status (overall analysis only). The correlation between BLyS mRNA levels and IFN-1 mRNA status at baseline was assessed using Spearman’s rank correlation coefficient. This was assessed further using a chi-square test to compare the subgroups. The correlation between BLyS protein levels and BLyS mRNA levels at baseline was also assessed using Spearman’s rank correlation coefficient. Time to first severe SELENA-SLEDAI Flare Index (SFI) flare over 52 weeks was compared between belimumab and placebo using a Cox proportional hazards model, with variables of the treatment group, study, baseline SELENA-SLEDAI score (≤ 9 versus ≥ 10), baseline proteinuria levels (< 2 g/24 h versus ≥ 2 g/24 h equivalent) and race (African descent or Native American descent versus other), and the BLyS subgroup or IFN-1 status (overall analysis only). This was carried out for BLyS mRNA tertiles (high, medium, low), BLyS mRNA revised subgroups (high, low), IFN-1 mRNA subgroups (high, low) and BLyS protein subgroups (high, low).

All statistical tests were 2-sided and performed at an overall significance level of 0.05.

## Results

### Baseline demographics and characteristics

A total of 554 patients were included in the gene expression population; of these, 281 had received belimumab and 273 had received placebo. Baseline demographics were generally similar across the BLyS and IFN-1 subgroups (Table 1); however, as expected, lower disease activity was seen in the BLyS mRNA/protein low and IFN-1 mRNA low subgroups compared with the other subgroups.

**Table 1:** Demographics and baseline characteristics

	BLyS mRNA low		BLyS mRNA medium		BLyS mRNA high		IFN-1 mRNA low		IFN-1 mRNA high		Revised BLyS mRNA low		Revised BLyS mRNA high		BLyS protein* low		BLyS protein* high	
	PBO	BEL	PBO	BEL	PBO	BEL	PBO	BEL	PBO	BEL	PBO	BEL	PBO	BEL	PBO	BEL	PBO	BEL
	*n* = 85	*n* = 99	*n* = 104	*n* = 81	*n* = 84	*n* = 101	*n* = 43	*n* = 49	*n* = 230	*n* = 232	*n* = 93	*n* = 111	*n* = 180	*n* = 170	*n* = 208	*n* = 220	*n* = 64	*n* = 61
Age, years, mean (SD)	39.2 (13.17)	38.8 (11.06)	36.5 (12.12)	36.8 (11.34)	37.6 (10.70)	37.0 (11.29)	43.3 (13.66)	42.5 (11.27)	36.6 (11.47)	36.5 (10.95)	38.9 (13.35)	38.5 (10.69)	37.0 (11.32)	36.9 (11.54)	38.0 (12.27)	38.4 (11.41)	36.3 (11.26)	34.4 (9.99)
Female, *n* (%)	79 (92.9)	96 (97.0)	95 (91.3)	81 (100.0)	78 (92.9)	97 (96.0)	39 (90.7)	47 (95.9)	213 (92.6)	227 (97.8)	86 (92.5)	108 (97.3)	166 (92.2)	166 (97.6)	197 (94.7)	213 (96.8)	54 (84.4)	61 (100.0)
SLE disease duration, years, mean (SD)	6.0 (6.23)	5.8 (7.30)	6.4 (6.41)	6.9 (6.54)	7.7 (6.35)	6.3 (5.99)	6.0 (6.52)	4.8 (5.99)	6.8 (6.32)	6.6 (6.72)	6.1 (6.07)	5.8 (7.12)	7.0 (6.48)	6.7 (6.28)	6.3 (6.09)	6.0 (6.38)	8.1 (7.03)	7.3 (7.41)
BILAG organ domain involvement, *n* (%)																		
≥ 1A or 2B	54 (63.5)	47 (47.5)	63 (60.6)	48 (59.3)	56 (66.7)	57 (56.4)	28 (65.1)	24 (49.0)	145 (63.0)	128 (55.2)	60 (64.5)	56 (50.5)	113 (62.8)	96 (56.5)	126 (60.6)	112 (50.9)	47 (73.4)	40 (65.6)
≥ 1A	8 (9.4)	3 (3.0)	10 (9.6)	12 (14.8)	13 (15.5)	9 (8.9)	6 (14.0)	1 (2.0)	25 (10.9)	23 (9.9)	8 (8.6)	3 (2.7)	23 (12.8)	21 (12.4)	25 (12.0)	18 (8.2)	6 (9.4)	6 (9.8)
≥ 1A or 1B	80 (94.1)	84 (84.8)	94 (90.4)	72 (88.9)	76 (90.5)	89 (88.1)	41 (95.3)	43 (87.8)	209 (90.9)	202 (87.1)	86 (92.5)	96 (86.5)	164 (91.1)	149 (87.6)	191 (91.8)	187 (85.0)	58 (90.6)	58 (95.1)
No A or B	5 (5.9)	15 (15.2)	10 (9.6)	9 (11.1)	8 (9.5)	12 (11.9)	2 (4.7)	6 (12.2)	21 (9.1)	30 (12.9)	7 (7.5)	15 (13.5)	16 (8.9)	21 (12.4)	17 (8.2)	33 (15.0)	6 (9.4)	3 (4.9)
SELENA-SLEDAI category, *n* (%)																		
≤ 9	55 (64.7)	56 (56.6)	51 (49.0)	35 (43.2)	35 (41.7)	48 (47.5)	27 (62.8)	33 (67.3)	114 (49.6)	106 (45.7)	57 (61.3)	59 (53.2)	84 (46.7)	80 (47.1)	117 (56.3)	118 (53.6)	24 (37.5)	21 (34.4)
10–11	19 (22.4)	20 (20.2)	27 (26.0)	20 (24.7)	19 (22.6)	22 (21.8)	9 (20.9)	9 (18.4)	56 (24.3)	53 (22.8)	22 (23.7)	23 (20.7)	43 (23.9)	39 (22.9)	47 (22.6)	46 (20.9)	17 (26.6)	16 (26.2)
≥ 12	11 (12.9)	23 (23.2)	26 (25.0)	26 (32.1)	30 (35.7)	31 (30.7)	7 (16.3)	7 (14.3)	60 (26.1)	73 (31.5)	14 (15.1)	29 (26.1)	53 (29.4)	51 (30.0)	44 (21.2)	56 (25.5)	23 (35.9)	24 (39.3)
SELENA-SLEDAI score, mean (SD)	8.6 (2.43)	9.2 (3.74)	9.4 (3.87)	9.6 (3.50)	10.4 (4.57)	9.7 (3.56)	8.6 (2.91)	8.5 (3.65)	9.6 (3.92)	9.7 (3.56)	8.8 (2.73)	9.3 (3.64)	9.8 (4.21)	9.6 (3.59)	8.9 (3.21)	9.2 (3.59)	11.0 (4.98)	10.5 (3.48)
Anti-dsDNA (≥ 30 IU/mL), *n* (%)	42 (49.4)	57 (57.6)	75 (72.1)	58 (71.6)	61 (72.6)	82 (81.2)	17 (39.5)	18 (36.7)	161 (70.0)	179 (77.2)	49 (52.7)	65 (58.6)	129 (71.7)	132 (77.6)	126 (60.6)	145 (65.9)	51 (79.7)	52 (85.2)
Low C3 and/or low C4, *n* (%)	39 (45.9)	62 (62.6)	71 (68.3)	52 (64.2)	61 (72.6)	75 (74.3)	12 (27.9)	21 (42.9)	159 (69.1)	168 (72.4)	44 (47.3)	69 (62.2)	127 (70.6)	120 (70.6)	121 (58.2)	137 (62.3)	49 (76.6)	52 (85.2)
Anti-dsDNA (≥ 30 IU/mL) and low C3 and/or low C4), *n* (%)	26 (30.6)	44 (44.4)	60 (57.7)	43 (53.1)	52 (61.9)	70 (69.3)	8 (18.6)	10 (20.4)	130 (56.5)	147 (63.4)	31 (33.3)	48 (43.2)	107 (59.4)	109 (64.1)	97 (46.6)	110 (50.0)	40 (62.5)	47 (77.0)

*One patient was excluded as he/she did not have a baseline BLyS protein assessment

*BEL* belimumab, *BILAG* British Isles Lupus Assessment Group of SLE Clinics, *BLyS* B lymphocyte stimulator, *IFN* interferon, *IFN-1* type 1 IFN-inducible gene signature, *mRNA* messenger ribonucleic acid, *PBO* placebo, *SD* standard deviation, *SELENA-SLEDAI* Safety of Estrogen in Lupus Erythematosus National Assessment trial-Systemic Lupus Erythematosus Disease Activity Index, *SLE* systemic lupus erythematosus

### BLyS and IFN-1 correlations

**Fig. 1:**
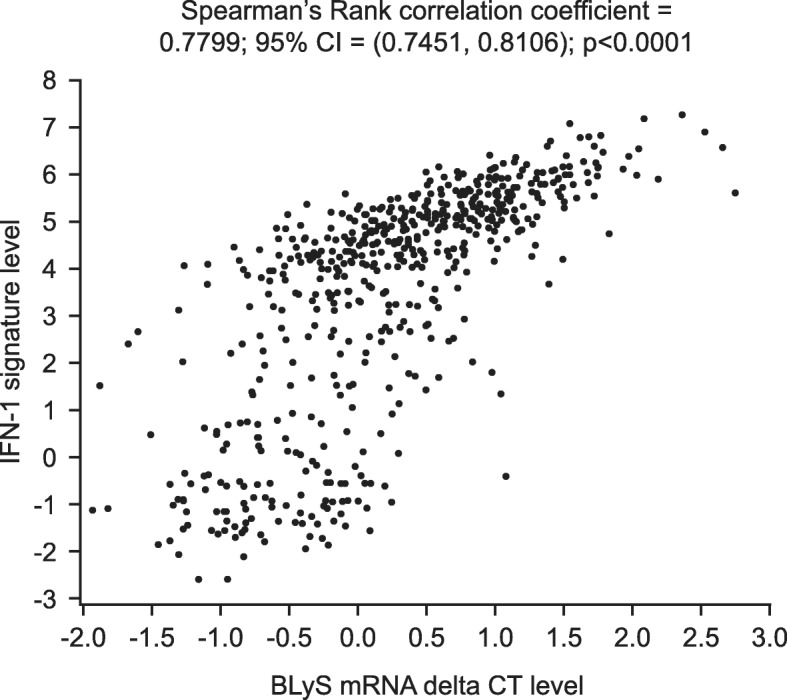
Correlation between baseline BLyS mRNA levels and baseline IFN-1 levels*.* One patient did not receive a dose of study medication but is included here as his/her baseline gene expression sample was analysed. BLyS B lymphocyte stimulator, CI confidence interval, CT cycle threshold, IFN interferon, IFN-1 type 1 IFN-inducible gene signature, mRNA messenger ribonucleic acid

Overall baseline BLyS mRNA levels and IFN-1 mRNA status were highly correlated (Spearman’s rank correlation coefficient 0.7799; 95% CI 0.7451, 0.8106; *p* < 0.0001 (Fig. 1, Additional file 3: Table S1)), confirmed with chi-square tests of BLyS mRNA tertiles (high/medium/low) and IFN-1 mRNA subgroups (high/low, *p* < 0.0001 (Additional file 4: Table S2)) and BLyS mRNA (high/low) and IFN-1 mRNA subgroups (high/low, *p* < 0.0001 (Additional file 5: Table S3)). BLyS protein and BLyS mRNA levels had a moderate correlation that was statistically significant due to the high sample size (Spearman’s rank correlation coefficient 0.2891; 95% CI 0.2108, 0.3636; *p* < 0.0001 (Additional file 2: Supplementary Figure S2, Additional file 6: Table S4)).

### SRI4 response to belimumab at week 52 of treatment

**Fig. 2:**
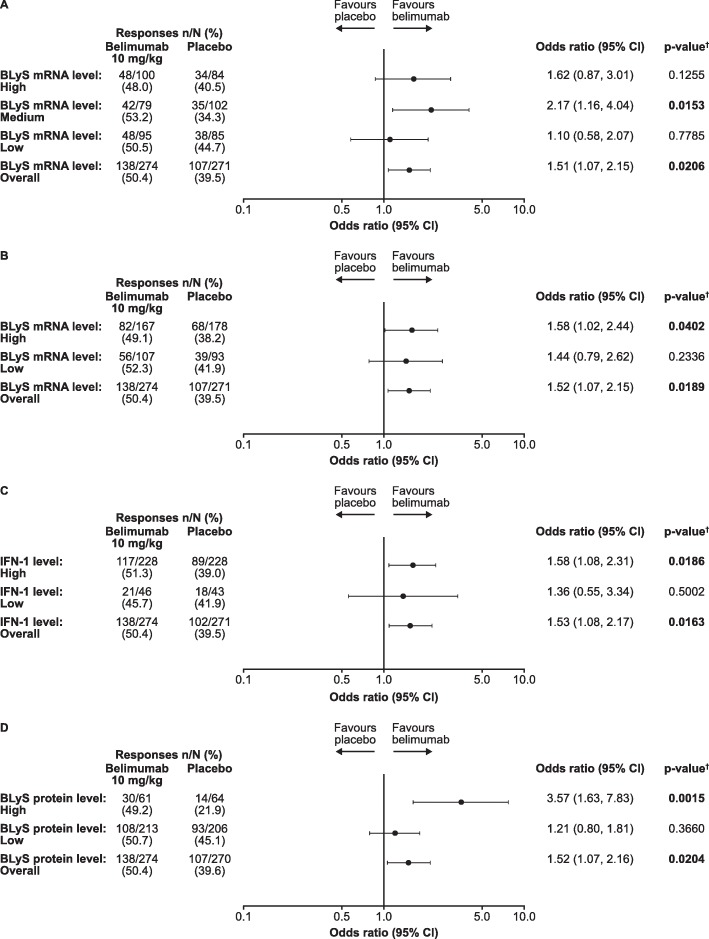
SRI4 response at week 52 of treatment*.* In patients with SELENA-SLEDAI ≥ 4 at baseline; ^†^covariates include the treatment group, study, baseline SELENA-SLEDAI score (≤ 9 versus ≥ 10), baseline proteinuria level (< 2 g/24 h versus ≥ 2 g/24 h equivalent) and race (African descent or Native American descent versus other). For the overall analysis, the BLyS mRNA/IFN-1/BLyS protein subgroup was included as an additional covariate. BLyS B lymphocyte stimulator, CI confidence interval, IFN interferon, IFN-1 type 1 IFN-inducible gene signature, mRNA messenger ribonucleic acid, SELENA-SLEDAI Safety of Estrogens in Lupus Erythematosus National Assessment-Systemic Lupus Erythematosus Disease Activity Index, SRI Systemic Lupus Erythematosus Responder Index

The percentage of SRI4 responders at week 52 was greater for the belimumab group compared with the placebo group for the low, medium and high BLyS mRNA subgroups (Fig. 2a). The odds ratio (OR) was not statistically significant for the low BLyS mRNA subgroup (OR 1.10; 95% CI 0.58, 2.07; *p* = 0.7785) or the high BLyS mRNA subgroup (OR 1.62; 95% CI 0.87, 3.01; *p* = 0.1255); however, it was statistically significant for the medium BLyS mRNA subgroup (OR 2.17; 95% CI 1.16, 4.04; *p* = 0.0153; Fig. 2a). When assessing based on binary stratification of BLyS mRNA levels (high/low), the proportion of SRI4 responders at week 52 was higher with belimumab versus placebo in both subgroups, but the difference reached significance in the high subgroup only (OR 1.58; 95% CI 1.02, 2.44; *p* = 0.0402 (Fig. 2b)). There were also more SRI4 responders at week 52 for belimumab versus placebo in both high and low IFN-1 mRNA subgroups, but the difference reached statistical significance in the high IFN-1 subgroup only (OR 1.58; 95% CI 1.08, 2.31; *p* = 0.0186; Fig. 2c). Likewise, the proportion of SRI4 responders at week 52 was higher with belimumab versus placebo in both BLyS protein level subgroups, but the difference reached significance in the high subgroup only (OR 3.57; 95% CI 1.63, 7.83; *p* = 0.0015 (Fig. 2d)).

**Fig. 3:**
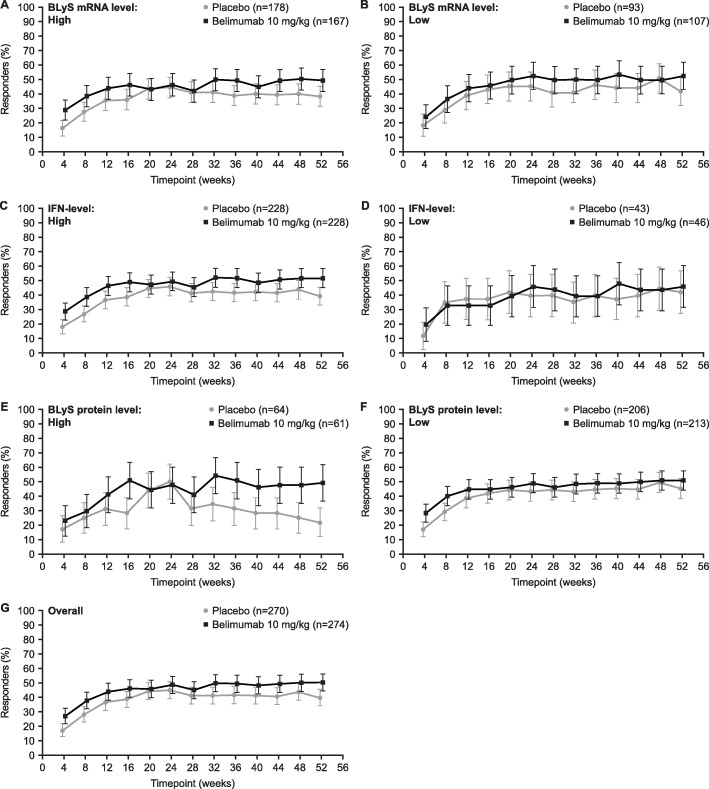
SRI4 response (in patients with SELENA-SLEDAI ≥ 4 at baseline) by visit*.* BLyS B lymphocyte stimulator, IFN interferon, IFN-1 type 1 IFN-inducible gene signature, mRNA messenger ribonucleic acid, SELENA-SLEDAI Safety of Estrogens in Lupus Erythematosus National Assessment-Systemic Lupus Erythematosus Disease Activity Index, SLE systemic lupus erythematosus, SRI Systemic Lupus Erythematosus Responder Index

There were more SRI4 responders in belimumab-treated versus placebo-treated patients at almost all timepoints in both the high and low revised BLyS mRNA subgroups (Fig. 3a, b). The proportion of SRI4 responders was higher with belimumab versus placebo at all timepoints in the IFN-1 high mRNA subgroup (Fig. 3c), but proportions were generally similar between belimumab and placebo in the IFN-1 low mRNA subgroup (Fig. 3d). In both the high and low BLyS protein level subgroups, the proportion of SRI4 responders was higher with belimumab than with placebo; however, this difference was greatest in the high BLyS protein subgroup (Fig. 3e, f). The overall response is also included for comparison (Fig. 3g).

### Time to first severe SFI flare over 52 weeks

**Fig. 4:**
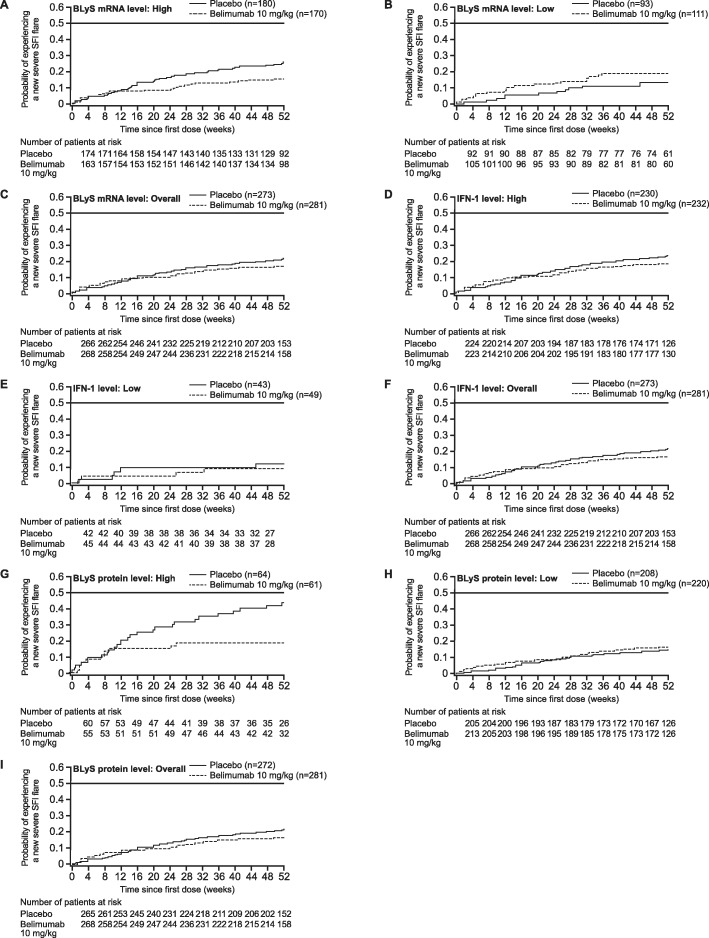
Time to first severe SFI flare over 52 weeks. BLyS B lymphocyte stimulator, IFN interferon, IFN-1 type 1 IFN-inducible gene signature, mRNA messenger ribonucleic acid, SELENA-SLEDAI Safety of Estrogens in Lupus Erythematosus National Assessment-Systemic Lupus Erythematosus Disease Activity Index, SFI SELENA-SLEDAI Flare Index

In the revised BLyS mRNA high subgroup, patients receiving belimumab had significantly lower risk of severe SFI flare than those receiving placebo (hazard ratio [HR] 0.59; 95% CI 0.36, 0.97; *p* = 0.0371; Additional file 7: Table S5). Although the risk of severe SFI flare was higher with belimumab versus placebo in the BLyS mRNA low subgroup, the difference did not reach statistical significance (HR 1.24; 95% CI 0.61, 2.51; *p* = 0.5576; Fig. 4a–c; Additional file 7: Table S5). The risk of severe SFI flare was lower in patients treated with belimumab versus those treated with placebo in both the IFN-1 low mRNA (HR 0.70; 95% CI 0.16, 3.07) and high (HR 0.75; 95% CI 0.50, 1.13) subgroups, but the difference did not reach statistical significance in either group (*p* = 0.6382 and *p* = 0.1716, respectively; Fig. 4d–f; Additional file 7: Table S5). The risk of severe SFI flare was significantly lower in belimumab- versus placebo-treated patients in the BLyS protein high subgroup (HR 0.39; 95% CI 0.19, 0.79; *p* = 0.0090), but not in the BLyS protein low subgroup (HR 1.06; 95% CI 0.65, 1.73; *p* = 0.8146; Fig. 4g–i; Additional file 7: Table S5).

### Alternative SRI4 response category responders at week 52

The proportion of patients achieving an alternative SRI4 response (SRI4 responder and [SELENA-SLEDAI total score ≤ 4 or clinical SELENA-SLEDAI total score ≤ 2]) was consistently higher with belimumab versus placebo in BLyS mRNA high and low subgroups, IFN-1 mRNA high and low subgroups and BLyS protein high and low subgroups. However, the difference reached statistical significance only in the BLyS mRNA high (OR [95% CI] 1.64 [1.04, 2.57]; *p* = 0.0322), IFN-1 mRNA high (OR [95% CI] 1.57 [1.06, 2.33]; *p* = 0.0236) and BLyS protein high (OR [95% CI] 3.89 [1.63, 9.30]; *p* = 0.0023) subgroups only (Additional file 8: Table S6).

### SRI8 responders at week 52

The proportions of patients achieving an SRI8 response were higher with belimumab versus placebo in both BLyS mRNA subgroups, both IFN-1 mRNA subgroups and both BLyS protein subgroups; but the difference reached statistical significance only in the BLyS mRNA low (OR [95% CI] 3.52 [1.25, 9.97]; *p* = 0.0176) and IFN-1 mRNA high (OR [95% CI] 1.87 [1.01, 3.47]; *p* = 0.0459) subgroups only (Additional file 9: Table S7).

## Discussion

The presence of elevated levels of BLyS mRNA and protein in patients with SLE is well established [5, 7, 18, 22]. This post hoc meta-analysis of two previous phase 3 SLE studies aimed to explore the relationship between BLyS mRNA levels and/or IFN-1 mRNA status and clinical responses to IV belimumab (10 mg/kg) treatment in the SLE population.

Our data suggested a tendency towards better response to belimumab in patients who had higher baseline BLyS mRNA/protein and/or IFN-1 mRNA levels. However, there are a number of limitations associated with this exploratory post hoc analysis, which should be considered when interpreting the results. First, the sample size was based simply on the number of patients who had an mRNA sample that had passed quality control; therefore, the BLyS mRNA subgroups were not powered to detect a clinically significant difference in SRI4 response rates. In addition, differences in SLE populations and subsequent levels of BLyS transcriptional activity in different populations are likely to result in variation in tertile cut-offs between studies. We looked to address this limitation by anchoring our subgroup boundaries to a defined population of healthy controls, which may be a useful reference in future studies of this nature. Finally, prospective studies will be needed to confirm the relationship between BLyS/IFN-1 and treatment response shown in this exploratory post hoc analysis.

Our original approach was to stratify patients into tertiles in line with the publication by Zollars et al., which demonstrated that higher levels of BLyS gene expression were associated with higher levels of clinical and serological SLE activity when patients were divided into low, medium and high BLyS mRNA groups [19]. Upon analysing the data from the current study, it became apparent that the results from this stratification did not align with the findings from the Zollars study, with the best week 52 SRI4 response to belimumab seen in the medium BLyS mRNA level subgroup and non-significant improvements with belimumab versus placebo in the high and low BLyS mRNA level subgroups. Given the previous evidence of a correlation between BLyS mRNA/protein levels and disease activity [17–19], we decided to analyse our data according to additional revised strata consisting of two subgroups of high and low BLyS mRNA expression. For this, BLyS mRNA levels from a population of healthy volunteers were utilised to determine a typical distribution of BLyS mRNA levels such that a level above normal may be defined. Notably, this method may be useful for future cohorts to ensure that any definition of high/low BLyS mRNA is set relative to a normal BLyS level.

With the subgroups defined by the revised BLyS mRNA cut-off levels, the proportion of SRI4 responders at week 52 was significantly greater with belimumab versus placebo in patients in the high BLyS mRNA subgroup. Furthermore, significantly greater proportions of SRI4 responders with belimumab versus placebo were seen in the high IFN-1 and high BLyS protein subgroups, with between-treatment differences of 12.3% and 27.3%, respectively, compared with respective differences of 3.8% and 5.6% in the low IFN-1 and low BLyS protein subgroups. The relationship between BLyS protein levels and response to belimumab was demonstrated in a previous post hoc analysis of the BLISS studies [17], and these data are in keeping with a prospective study that demonstrated greater disease activity with higher levels of BLyS protein in patients with SLE [6].

Secondary endpoint outcomes built upon the SRI4 responder findings, with patients in the high BLyS mRNA and high BLyS protein subgroups also demonstrating significantly lower risk of severe SFI flare with belimumab versus placebo. Furthermore, the alternative SRI4 response (SRI4 responder and [SELENA-SLEDAI total score ≤ 4 or clinical SELENA-SLEDAI total score ≤ 2]) was consistently achieved by higher proportions of belimumab-treated than placebo-treated patients, but differences only reached significance in the high BLyS mRNA, high IFN-1 mRNA and high BLyS protein subgroups. The proportion of SRI8 responders with belimumab versus placebo was also significantly higher in the high IFN-1 mRNA subgroup. In contrast to other endpoints, the proportion of SRI8 responders was higher in the BLyS mRNA low subgroup. Although the reasons for a higher response in the BLyS mRNA low subgroup are not clear, it should be noted that there were very few SRI8 responders; therefore, the analysis is of limited utility. Nonetheless, taken together, our data indicate that the greatest response to belimumab is seen in patients with the high or medium BLyS mRNA expression, high IFN-1 mRNA expression and high BLyS protein levels at baseline. In contrast, proportions of patients treated with placebo plus standard of care treatments achieving an SRI4 response were similar in the low versus high BLyS mRNA (41.9% versus 38.2%) and IFN-1 mRNA level subgroups (41.9% versus 39.0%), and higher in the low versus high BLyS protein level subgroups (45.1% versus 21.9%). However, it is still important to note that patients from the low BLyS mRNA/protein and low IFN-1 mRNA level subgroups treated with belimumab still responded proportionally better than those treated with placebo, with between-treatment differences of 10.4%, 5.6% and 3.8%, respectively.

One key element of our study was exploration of the relationship between BLyS, IFN-1 and responses to belimumab in the SLE population. As may be expected, given the association between BLyS and IFN-1 [20, 21], baseline levels of BLyS mRNA and IFN-1 mRNA expression levels correlated well with each another. Additionally, BLyS mRNA and protein levels were moderately correlated. Of interest, although significantly greater responses to belimumab were shown with high BLyS mRNA, high IFN-1 mRNA and high BLyS protein, the response to belimumab versus placebo appeared to be greater in the high BLyS protein subgroup than in the high BLyS mRNA subgroup. This effect was seen in the SRI4 responder endpoint, time to first severe flare and the alternative SRI4 response category. These data may suggest that BLyS protein is a better predictor of clinical response than BLyS mRNA, consistent with the mode of action of belimumab, which directly targets the BLyS protein and inhibits its biological activity. Future studies to ascertain whether BLyS protein and IFN-1 mRNA levels are correlated would be of interest.

## Conclusions

The results from this post hoc meta-analysis suggest a tendency towards a better response to IV belimumab (10 mg/kg) as add-on therapy versus SoC alone in patients with high baseline BLyS protein and IFN-1 mRNA levels and medium/high BLyS mRNA levels. Additional studies into how these biomarkers correlate with each other and relate to treatment responses will be useful to inform future treatment decisions.

## Supplementary information

**Additional file 1: Supplementary Figure S1.** Distribution of biomarkers.

**Additional file 2: Supplementary Figure S2.** Correlation between BLyS protein and mRNA levels.

**Additional file 3: Table S1.** Correlation between BLyS mRNA and IFN-1 mRNA levels at baseline.

**Additional file 4: Table S2.** Cross tabulation of BLyS mRNA subgroups (tertiles) and IFN-1 mRNA subgroups.

**Additional file 5: Table S3.** Cross tabulation of revised BLyS mRNA subgroups (high/low) and IFN-1 mRNA subgroups (high/low).

**Additional file 6: Table S4.** Correlation between BLyS protein levels and BLyS mRNA levels.

**Additional file 7: Table S5.** Time to first severe SFI flare over 52 weeks.

**Additional file 8: Table S6.** Alternative response category responders at Week 52.

**Additional file 9: Table S7.** SRI-8 response at Week 52.

## Data Availability

Anonymised individual participant data and study documents can be requested for further research from www.clinicalstudydatarequest.com.

## Abbreviations

BEL: Belimumab
BILAG: British Isles Lupus Assessment Group of SLE Clinics
BLyS: B lymphocyte stimulator
CI: Confidence interval
CT: Cycle threshold
HR: Hazard ratio
IFN: Interferon
IFN-1: Type 1 IFN-inducible gene signature
IV: Intravenous
mRNA: Messenger ribonucleic acid
OR: Odds ratio
PBO: Placebo
PGA: PHYSICIAN Global Assessment
SD: Standard deviation
SELENA-SLEDAI: Safety of Estrogen in Lupus Erythematosus National Assessment-Systemic Lupus Erythematosus Disease Activity Index
SFI: SELENA-SLEDAI Flare Index
SLE: Systemic lupus erythematosus
SoC: Standard of care
SRI: Systemic Lupus Erythematosus Responder Index
