# Supplementary material for: The role of baseline BLyS levels and type 1 interferon-inducible gene signature status in determining belimumab response in systemic lupus erythematosus: a post hoc meta-analysis
Source: Arthritis Res Ther. 2020 May 4;22:102. doi: 10.1186/s13075-020-02177-0 (PMC7197114; doi:10.1186/s13075-020-02177-0)
Supplement: Supplementary file 3 — Additional file 3: Table S1. Correlation between BLyS mRNA and IFN-1 mRNA levels at baseline. [file 13075_2020_2177_MOESM3_ESM.docx]

**Table S1: Correlation between BLyS mRNA and IFN-1 mRNA levels at baseline***

|  | **BLyS mRNA**  **(N=555)** | **IFN-1 mRNA**^†^  **(N=555)** |
| --- | --- | --- |
| Mean (SD) | 0.287 (0.7941) | 3.735 (2.2732) |
| Spearman’s rank correlation coefficient (95% CI) | 0.7799  (0.7451, 0.8106) | |
| p-value | <0.0001 | |

*One patient did not receive a dose of study medication but is included here as their baseline gene expression sample was analysed; ^†^summary statistics for the IFN signature levels should be interpreted with caution due to the distribution of the data

BLyS: B-lymphocyte stimulator; CI: confidence interval; IFN: interferon; IFN-1: type 1 IFN-inducible gene signature; mRNA: messenger ribonucleic acid; SD: standard deviation
