# Supplementary material for: The role of baseline BLyS levels and type 1 interferon-inducible gene signature status in determining belimumab response in systemic lupus erythematosus: a post hoc meta-analysis
Source: Arthritis Res Ther. 2020 May 4;22:102. doi: 10.1186/s13075-020-02177-0 (PMC7197114; doi:10.1186/s13075-020-02177-0)
Supplement: Supplementary file 4 — Additional file 4: Table S2. Cross tabulation of BLyS mRNA subgroups (tertiles) and IFN-1 mRNA subgroups. [file 13075_2020_2177_MOESM4_ESM.docx]

**Table S2: Cross tabulation of BLyS mRNA subgroups (tertiles) and IFN-1 mRNA subgroups***

| **BLyS mRNA subgroup** | **N** | **IFN-1 mRNA**  **Low**  **(n=92)** | **IFN-1 mRNA**  **High**  **(n=463)** |
| --- | --- | --- | --- |
| Low | 185 | 78 (42.2%) | 107 (57.8%) |
| Medium | 185 | 13 (7.0%) | 172 (93.0%) |
| High | 185 | 1 (0.5%) | 184 (99.5%) |
| p-value^†^ | <0.0001 | | |

*One patient did not receive a dose of study medication but is included here as their baseline gene expression sample was analysed; ^†^using a chi-square test

BLyS: B-lymphocyte stimulator; IFN: interferon; IFN-1: type 1 IFN-inducible gene signature; mRNA: messenger ribonucleic acid
