# Supplementary material for: The role of baseline BLyS levels and type 1 interferon-inducible gene signature status in determining belimumab response in systemic lupus erythematosus: a post hoc meta-analysis
Source: Arthritis Res Ther. 2020 May 4;22:102. doi: 10.1186/s13075-020-02177-0 (PMC7197114; doi:10.1186/s13075-020-02177-0)
Supplement: Supplementary file 5 — Additional file 5: Table S3. Cross tabulation of revised BLyS mRNA subgroups (high/low) and IFN-1 mRNA subgroups (high/low). [file 13075_2020_2177_MOESM5_ESM.docx]

## Table S3: Cross tabulation of BLyS mRNA subgroups (high/low) and IFN-1 mRNA subgroups (high/low)*

| **BLyS mRNA subgroup** | **n** | **IFN-1 mRNA**  **Low**  **(n=92)** | **IFN-1 mRNA**  **High**  **(n=463)** |
| --- | --- | --- | --- |
| Low | 205 | 81 (39.5%) | 124 (60.5%) |
| High | 350 | 11 (3.1%) | 339 (96.9%) |
| p-value^†^ | <0.0001 | | |

*One patient did not receive a dose of study medication but is included here as their baseline gene expression sample was analysed; ^†^using a chi-square test

BLyS: B-lymphocyte stimulator; IFN: interferon; IFN-1: type 1 IFN-inducible gene signature; mRNA: messenger ribonucleic acid
