# Supplementary material for: The role of baseline BLyS levels and type 1 interferon-inducible gene signature status in determining belimumab response in systemic lupus erythematosus: a post hoc meta-analysis
Source: Arthritis Res Ther. 2020 May 4;22:102. doi: 10.1186/s13075-020-02177-0 (PMC7197114; doi:10.1186/s13075-020-02177-0)
Supplement: Supplementary file 6 — Additional file 6: Table S4. Correlation between BLyS protein levels and BLyS mRNA levels. [file 13075_2020_2177_MOESM6_ESM.docx]

**Table S4**: **Correlation between BLyS protein levels and BLyS mRNA levels*^†^**

|  | **BLyS protein**  **(N=554*)** | **BLyS mRNA**  **(N=554*)** |
| --- | --- | --- |
| Mean (SD) | 1.659 (1.4225) | 0.286 (0.7948) |
| Spearman’s rank correlation coefficient (95% CI) | 0.2891  (0.2108, 0.3636) | |
| p-value | <0.0001 | |

*One patient did not receive a dose of study medication but is included here as their baseline gene expression sample was analysed; ^†^one patient was excluded as they did not have a baseline BLyS protein assessment

BLyS: B-lymphocyte stimulator; CI: confidence interval; mRNA: messenger ribonucleic acid; SD: standard deviation
