# Supplementary material for: The role of baseline BLyS levels and type 1 interferon-inducible gene signature status in determining belimumab response in systemic lupus erythematosus: a post hoc meta-analysis
Source: Arthritis Res Ther. 2020 May 4;22:102. doi: 10.1186/s13075-020-02177-0 (PMC7197114; doi:10.1186/s13075-020-02177-0)
Supplement: Supplementary file 7 — Additional file 7: Table S5. Time to first severe SFI flare over 52 weeks. [file 13075_2020_2177_MOESM7_ESM.docx]

## Table S5: Time to first severe SFI flare over 52 weeks

|  | **Revised BLyS mRNA Low** | | **Revised BLyS mRNA High** | | **IFN-1 mRNA**  **Low** | | **IFN-1 mRNA**  **High** | | **BLyS protein Low** | | **BLyS protein High** | |
| --- | --- | --- | --- | --- | --- | --- | --- | --- | --- | --- | --- | --- |
| **Time to first severe SFI flare over 52 weeks** | | | | | | | | | | | | |
| Population | PBO  (n=93) | BEL  (n=111) | PBO  (n=180) | BEL  (n=170) | PBO  (n=43) | BEL  (n=49) | PBO  (n=230) | BEL  (n=232) | PBO  (n=208) | BEL  (n=220) | PBO  (n=64) | BEL  (n=61) |
| Patients, n (%) | 13 (14.0) | 20 (18.0) | 45 (25.0) | 25 (14.7) | 5 (11.6) | 4 (8.2) | 53 (23.0) | 41 (17.7) | 31 (14.9) | 34 (15.5) | 27 (42.2) | 11 (18.0) |
| Hazard ratio  (95% CI)  BEL versus PBO* | 1.24 (0.61, 2.51) | | 0.59 (0.36, 0.97) | | 0.70 (0.16, 3.07) | | 0.75 (0.50, 1.13) | | 1.06 (0.65, 1.73) | | 0.39 (0.19, 0.79) | |
| p-value* | 0.5576 | | 0.0371 | | 0.6382 | | 0.1716 | | 0.8146 | | 0.0090 | |

BEL: belimumab; BLyS: B-lymphocyte stimulator; CI: confidence interval; IFN: interferon; IFN-1: type 1 IFN-inducible gene signature; mRNA: messenger ribonucleic acid; PBO: placebo; SELENA-SLEDAI: Safety of Estrogen in Lupus Erythematosus National Assessment-Systemic Lupus Erythematosus Disease Activity Index; SFI: SELENA-SLEDAI Flare Index; SRI: Systemic Lupus Erythematosus Responder Index

*Covariates include treatment group, study, baseline SELENA-SLEDAI score (≤9 versus ≥10), baseline proteinuria (<2 g/24 versus ≥2 g/24 h equivalent) and race (African descent or indigenous American descent versus other)
