# Supplementary material for: The role of baseline BLyS levels and type 1 interferon-inducible gene signature status in determining belimumab response in systemic lupus erythematosus: a post hoc meta-analysis
Source: Arthritis Res Ther. 2020 May 4;22:102. doi: 10.1186/s13075-020-02177-0 (PMC7197114; doi:10.1186/s13075-020-02177-0)
Supplement: Supplementary file 8 — Additional file 8: Table S6. Alternative response category responders at Week 52. [file 13075_2020_2177_MOESM8_ESM.docx]

## Table S6: Alternative response category responders at Week 52

|  | **Revised BLyS mRNA Low** | | **Revised BLyS mRNA High** | | **IFN-1 mRNA**  **Low** | | **IFN-1 mRNA**  **High** | | **BLyS protein Low** | | **BLyS protein High** | |
| --- | --- | --- | --- | --- | --- | --- | --- | --- | --- | --- | --- | --- |
| **Alternative response: SRI-4 responder AND (SELENA-SLEDAI total score ≤4 OR clinical SELENA-SLEDAI total score ≤2) among patients with SELENA-SLEDAI ≥4 at baseline** | | | | | | | | | | | | |
| Population | PBO  (n=93) | BEL  (n=107) | PBO  (n=178) | BEL  (n=167) | PBO  (n=43) | BEL  (n=46) | PBO  (n=228) | BEL  (n=228) | PBO  (n=206) | BEL  (n=213) | PBO  (n=64) | BEL  (n=61) |
| Responders, n (%) | 32 (34.4) | 43 (40.2) | 51 (28.7) | 67 (40.1) | 15 (34.9) | 17 (37.0) | 68 (29.8) | 93 (40.8) | 73 (35.4) | 86 (40.4) | 10 (15.6) | 24 (39.3) |
| Odds ratio  (95% CI)  BEL versus PBO* | 1.31 (0.71, 2.40) | | 1.64 (1.04, 2.57) | | 1.08 (0.43, 2.75) | | 1.57 (1.06, 2.33) | | 1.20 (0.80, 1.79) | | 3.89 (1.63, 9.30) | |
| p-value* | 0.3897 | | 0.0322 | | 0.8648 | | 0.0236 | | 0.3840 | | 0.0023 | |

*Covariates include treatment group, study, baseline SELENA-SLEDAI score (≤9 versus ≥10), baseline proteinuria (<2 g/24 h versus ≥2 g/24 h equivalent) and race (African descent or Native American descent versus other)

BEL: belimumab; BLyS: B-lymphocyte stimulator; CI: confidence interval; IFN: interferon; IFN-1: type 1 IFN-inducible gene signature; mRNA: messenger ribonucleic acid; PBO: placebo; SELENA-SLEDAI: Safety of Estrogens in Lupus Erythematosus National Assessment-Systemic Lupus Erythematosus Disease Activity Index; SRI: Systemic Lupus Erythematosus Responder Index
