# Supplementary material for: The role of baseline BLyS levels and type 1 interferon-inducible gene signature status in determining belimumab response in systemic lupus erythematosus: a post hoc meta-analysis
Source: Arthritis Res Ther. 2020 May 4;22:102. doi: 10.1186/s13075-020-02177-0 (PMC7197114; doi:10.1186/s13075-020-02177-0)
Supplement: Supplementary file 9 — Additional file 9: Table S7. SRI-8 response at Week 52. [file 13075_2020_2177_MOESM9_ESM.docx]

## Table S7: SRI-8 response at Week 52

|  | **Revised BLyS mRNA**  **Low** | | **Revised BLyS mRNA High** | | **IFN-1 mRNA**  **Low** | | **IFN-1 mRNA**  **High** | | **BLyS protein Low** | | **BLyS protein High** | |
| --- | --- | --- | --- | --- | --- | --- | --- | --- | --- | --- | --- | --- |
| **SRI-8 response among patients with SELENA-SLEDAI ≥8 at baseline** | | | | | | | | | | | | |
| Population | PBO  (n=71) | BEL  (n=85) | PBO  (n=131) | BEL  (n=127) | PBO  (n=30) | BEL  (n=37) | PBO  (n=172) | BEL  (n=175) | PBO  (n=150) | BEL  (n=161) | PBO  (n=51) | BEL  (n=51) |
| Responders, n (%) | 6  (8.5) | 22  (25.9) | 19  (14.5) | 21  (16.5) | 4  (13.3) | 6  (16.2) | 21 (12.2) | 37 (21.1) | 22 (14.7) | 35 (21.7) | 3  (5.9) | 8  (15.7) |
| Odds ratio  (95% CI) BEL versus PBO* | 3.52 (1.25, 9.97) | | 1.32 (0.65, 2.69) | | 2.25 (0.47, 10.82) | | 1.87 (1.01, 3.47) | | 1.76 (0.96, 3.24) | | 3.24 (0.78, 13.50) | |
| p-value* | 0.0176 | | 0.4402 | | 0.3110 | | 0.0459 | | 0.0696 | | 0.1070 | |

*Covariates include treatment group, study, baseline SELENA-SLEDAI score (≤9 versus ≥10), baseline proteinuria (<2 g/24 h versus ≥2 g/24 h equivalent) and race (African descent or Native American descent versus other). Note that baseline SELENA-SLEDAI score was removed from the BLyS protein subgroup analysis due to low cell counts

BLyS: B-lymphocyte stimulator; BEL: belimumab; CI: confidence interval; IFN: interferon; IFN-1: type 1 IFN-inducible gene signature; mRNA: messenger ribonucleic acid; PBO: placebo; SELENA-SLEDAI: Safety of Estrogens in Lupus Erythematosus National Assessment - Systemic Lupus Erythematosus Disease Activity Index; SRI: Systemic Lupus Erythematosus Responder Index
